# Technostress in Spain between 2016 and 2024: perception of the impact of teleworking on the health of Spanish workers

**DOI:** 10.3389/fpsyg.2026.1774427

**Published:** 2026-03-04

**Authors:** Agustin Sánchez-Toledo Ledesma

**Affiliations:** Universidad Internacional de La Rioja (UNIR), Logroño, Spain

**Keywords:** technostress, teleworking, ICT, occupational health, psychosocial risks

## Abstract

**Background:**

The COVID-19 pandemic accelerated the use of information and communication technologies (ICT) in work settings, raising concerns about technostress and its potential impact on workers’ health and performance.

**Objective:**

To compare technostress-related assessments associated with ICT use among Spanish teleworkers in 2016 and 2024, identifying changes in perceptions and perceived impacts.

**Design:**

Retrospective, observational, quantitative, comparative study using two independent cross-sectional samples.

**Methods:**

A total of 758 Spanish teleworkers completed an online validated questionnaire in 2016 and 2024. Group differences were examined using chi-square tests [with Cramer’s V/*φ* and odds ratios (OR) with 95% confidence intervals (CI) for dichotomous outcomes], and independent-samples comparisons (*t*-test and Mann–Whitney *U* with Rosenthal’s *r* as effect size).

**Results:**

ICT use for family and leisure purposes was lower in 2024, although associations were small [family: *φ* = 0.076, OR = 1.38, 95% CI (1.02, 1.88); leisure: φ = 0.095, OR = 1.58, 95% CI (1.12, 2.22)]. Reports that social networks and mobile phones caused problems increased modestly (social networks: *V* = 0.138; mobile phones: *V* = 0.121). Ratings of personal experience with ICT shifted significantly (*p* < 0.001) with the largest association observed in the study (*V* = 0.215). Regarding technostress subscales, perceived autonomy and positive consequences were lower in 2024 (both *p* = 0.002; |*r*| ≈ 0.14), whereas negative consequences (*p* < 0.001; |*r*| ≈ 0.21) and perceived capacity to work well using ICT (*p* = 0.014; |*r*| ≈ 0.11) were higher in 2024. Overall, effects were generally small in magnitude but consistent.

**Conclusion:**

Between 2016 and 2024, Spanish teleworkers showed statistically significant but mostly small changes in technostress-related perceptions. The most consistent pattern was a modest increase in perceived negative consequences alongside slight gains in perceived capacity to manage ICT-related demands. These findings support the need for preventive occupational policies that support healthy teleworking conditions.

## 1 Introduction

Nowadays, the widespread use of Information and Communication Technologies (ICT) is a reality across almost all productive sectors. Owing to the capacity to connect individuals across geographical barriers, teleworking has emerged and grown significantly. This modality is characterized by the essential use of ICT. Employees performing their duties from home or another location agreed upon with their employer (Cárdenas and Bracho, 2020; Martín Rodriguez, 2020; Villagómez and Ramírez, 2022).

Most labor research coincides in highlighting that the number of teleworkers increased following the health restrictions resulting from the COVID-19 pandemic (Anghel et al., 2020; Peiró and Soler, 2020; Molina et al., 2021; Observatorio Nacional de Tecnología y Sociedad, 2021; Benavides and Silva-Peñaherrera, 2022). Official Spanish data show that teleworking increased from 8.3% in late 2019 to 19.1% in mid-2020. During the pandemic, it remained between 14.5% and 16.4%, and it has since stabilized at around 14.4% in early 2024.

A similar surge occurred globally. In Latin America, over 23 million people began teleworking in 2020, with 20%–30% working from home during the pandemic (International Labour Organization, 2021). In Europe, before 2019, only four countries had over 10% of teleworkers; this later increased to more than half of European countries, consolidating telework in the labor market.

Various benefits of teleworking have been identified, including shorter working times, greater autonomy, and improved responsibility among teleworkers, potentially leading to productivity gains. Nevertheless, it is also recognized that working from home exposes teleworkers to occupational safety and health risks similar to those present in traditional workplaces, notably psychosocial and ergonomic risks (Cárdenas and Bracho, 2020).

This study focuses on technostress as a direct consequence of ICT use at work. Technostress refers to stress or a negative psychological state caused by ICT use. Its risk factors include mental overload, multitasking without sufficient control, excessive work pace acceleration, and extended working hours beyond legal or contractual limits (Cárdenas and Bracho, 2020; Martín Rodriguez, 2020). People who telework are particularly susceptible to this condition (Vivas-Manrique et al., 2022; Villavicencio-Ayub et al., 2024).

In Spain, there are no official data or programmes specifically gathering information on technostress among teleworkers. Only independent studies have evaluated this condition in workplace contexts (Bustamante-Lozano and Torres-Jerves, 2024), and some studies have focused on academic environments (Penado-Abilleira et al., 2021; Ramos, 2022). Literature reviews have also aimed to clarify its concept and forms (Cuervo Carabel et al., 2018; Martín Rodriguez, 2020). Fortunately, the Technostress Observatory, a non-governmental initiative of the Institute for Occupational Safety and Well-being (ISBL, Spanish abbreviation), has collected data from various countries on the health effects of technostress among workers. There are two editions: 2016 and 2024 (Instituto de Seguridad y Bienestar Laboral, 2024). These data are valuable as they provide a first approximation of the technostress situation in some Spanish regions, enabling, for the first time, a multi centre comparative study on this topic. The scientific literature shows that research on technostress is still in an early stage; however, the available studies on the topic remain limited in scope, as they generally examine this phenomenon in a single group of people, one region, only one company or productive sector, and at just one point in time. This situation may be explained by the characteristics of the population under study: teleworkers are difficult to contact for data collection because they work from home most of the time and rarely gather in a single place.

In this context, a study such as the present one, which gathers data from teleworkers in different regions of Spain at two distinct points in time (2016 and 2024), makes it possible to identify changes in how they telework, their perceptions of this work modality, and the specific impacts they experience when they are stressed by their work. These changes are even more relevant considering that, between the two study periods, the COVID-19 pandemic occurred and, as previously noted, led to an increase in the work-related use of ICTs.

### 1.1 Current study

The present study aimed to compare technostress assessments among Spanish teleworkers in 2016 and 2024 using data from the Technostress Observatory. Telework has become a well-established form of work in today’s labor market, especially after the public health restrictions imposed during the COVID-19 pandemic, which led to an increase in the number of teleworkers. In this context, technostress has become a subject of research and, in the literature, it refers to workers’ stress that is a direct consequence of the ICT use (Cárdenas and Bracho, 2020; International Labour Organization, 2021; Vivas-Manrique et al., 2022; Villavicencio-Ayub et al., 2024). Therefore, this study is based on the following idea: workers are increasingly accustomed to ICT use, particularly due to the rise of remote work during the pandemic; nevertheless, their use may be associated with more negative consequences related to technostress.

**Figure 1:**
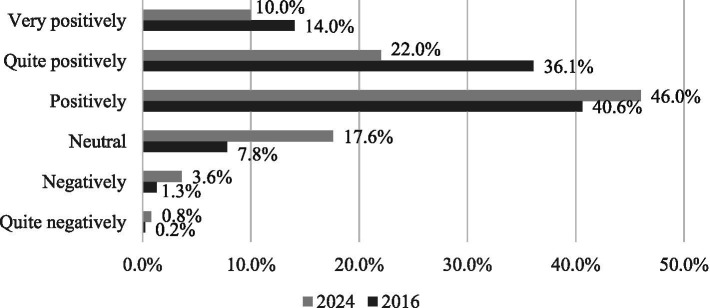
Distribution of Spanish teleworkers’ self-evaluation of their personal experience with ICT (2016 *vs.* 2024). This figure shows a decline in “very positive” and “quite positive” evaluations, alongside a change in “neutral” and “negative” responses from 2016 to 2024. Readers should note the marked rise in neutral perceptions and the drop in “quite positive” ratings, suggesting a more critical stance towards ICT use among teleworkers. Changes were statistically significant based on the chi-square test (*p* < 0.001, Cramer’s *V* = 0.215).

Building on this idea, a study was designed to compare changes in personal attitudes, implications, negative and positive consequences, autonomy, and ability to work with ICT, using data from the Technostress Observatory. These data cover a broad observation period, from 2016 (before the pandemic) through 2024 (after the pandemic). To assess technostress, the following dimensions were considered: negative implications of ICT use, autonomy in ICT use, negative consequences of ICT use, and positive consequences of ICT use, capacity to work well using ICT, positive personal posture, and negative personal posture. Based on these dimensions, the following hypothesis was proposed: between 2016 and 2024, an increase in workers’ capacity to use ICTs is expected, but also in the negative implications, consequences, and personal postures related to their use, together with a reduction in autonomy, positive personal posture, and the positive consequences of working with ICTs (see Figure 1).

## 2 Materials and methods

### 2.1 Study design

An empirical, retrospective, observational, quantitative, and comparative study with two cross-sectional assessments was designed. The study population consisted of Spanish workers engaged in teleworking. Data were obtained from the questionnaire administered in the second and third editions of the Technostress Observatory by the ISBL, after a prior request and with the informed consent of each participant. These data included factors and impacts related to technostress arising from ICT use, corresponding to the years 2016 and 2024.

### 2.2 Sample

The questionnaire was not administered to the same population of workers on two different occasions, but rather to the workers who took part in each of the two editions of the Technostress Observatory. Therefore, the present study is a comparative cross-sectional study based on independent samples and focuses on changes or differences in trends at the worker population level, rather than on the evolution of the variables under study over time.

According to information provided by the ISBL, there was an approximate participation of 3,000 workers across both editions. Workers from various productive sectors, from any region in Spain, who teleworked at least three or more days per week were included. Temporary workers or those who did not complete full working days were excluded.

The sample size was calculated using a probabilistic sampling formula based on a normal distribution. Considering a 95% confidence level, a sample of 1,067 workers was obtained. Subsequently, an adjustment for finite population was applied. In order to manage potential biases derived from the cross-sectional design and differences between the 2016 and 2024 samples. The final sample size was determined by the number of eligible respondents available in each survey wave (total *n* = 758). Because the study is observational and the sample size was therefore not set *a priori*, we conducted an effect-size sensitivity analysis in G*Power (version 3.1) for two independent groups (Wilcoxon–Mann–Whitney test; two-tailed *α* = 0.05). With group sizes of *n* = 488 (2016) and *n* = 270 (2024), the design provides 80% power to detect a minimum standardized difference of approximately *d* ≈ 0.21, indicating adequate sensitivity to detect small between-group effects. Accordingly, results are interpreted primarily using effect sizes and 95% confidence intervals rather than statistical significance alone. Valid sample sizes vary slightly across analyses due to item-level missing data and are reported in each table.

### 2.3 Data collection technique and instrument

The information was collected using an online questionnaire designed by the institute, with direct invitations sent to employees. Subsequently, workers who had registered with the ISBL Technostress Observatory and had provided contact information were randomly selected. The selected workers were informed about the objectives of the study, what their participation would involve, and that their information would be treated confidentially and used only for the purposes of the study. Those workers who agreed to participate and from whom informed consent was obtained were included.

The “Questionnaire of Factors and Effects of Technostress in Teleworkers,” previously validated in a pilot group, was applied. This instrument allows for the investigation of patterns of ICT use and the problems these cause in workers’ daily lives through their self-perceptions. During validation, Cronbach’s alpha for each subscale ranged from 0.709 to 0.946, demonstrating internal consistency. The results of Bartlett’s sphericity test and the Kaiser-Meyer-Olkin (KMO) index determined that the subscales were suitable for factor analysis. In the KMO test, values ranged from 0.500 to 0.937; values above 0.700 were considered good, and the rest acceptable. Bartlett’s test produced a significance value of *p* < 0.001, indicating adequacy for factor analysis. Following an orthogonal VARIMAX rotation factor analysis, seven final subscales were established.

The first subscale comprised 18 questions regarding the implications of ICT in workload and work organization. It included questions related to time pressure, task overload and disorganization, and cognitive demand. The second subscale included three questions assessing the level of autonomy during telework, such as the ability to organize one’s own work, decide when to start or finish tasks, and make decisions when faced with unforeseen events. The third subscale consisted of two questions focusing on concerns about ICT use and the time consumed by these technologies. The fourth subscale included three questions on workers’ ability to balance family and work aspects and to manage their time better. The fifth subscale included five questions related to workers’ capacity to use ICT despite difficulties such as unexpected situations, technical failures, or the constant need for ICT updates. The sixth subscale comprised 28 questions exploring the positive aspects of teleworking from workers’ perspectives. The final subscale comprised 25 questions on the various negative aspects of teleworking. [Insert Table 1 here].

**Table 1:** Psychometric validation of the technostress factors and effects questionnaire in teleworkers (Cronbach’s *α*, KMO, Bartlett’s test, and number of items per subscale).

Dimension	Cronbach’s α	KMO	Barlett	Q1	Q2	Q3	Items
Negative implications of ICT use	0.934	0.922	< 0.001	32.25	48.00	64.00	18
Autonomy in ICT use	0.838	0.718	< 0.001	11.00	14.00	17.00	3
Negative consequences of ICT use	0.709	0.500	< 0.001	3.00	5.00	7.00	2
Positive consequences of ICT use	0.740	0.683	< 0.001	3.00	6.00	6.00	3
Capacity to work well using ICT	0.903	0.874	< 0.001	14.00	15.00	21.00	5
Positive personal posture towards ICT work	0.946	0.937	< 0.001	81.00	100.00	117.00	28
Negative personal posture towards ICT work	0.924	0.876	< 0.001	36.00	55.00	73.25	25

KMO, Kaiser-Meyer-Olkin test; Q, quartile; ICT, Information and communication technologies.

Each of the above subscales was determined based on a series of five-point Likert-type questions. Scores were assigned to each response option. The final score for each variable corresponded to the sum of scores obtained across questions, divided by the total number of questions. Thus, for the variable “negative implications of ICT use,” higher scores reflected modest increase implications; for “autonomy in ICT use,” higher scores indicated greater autonomy; for “negative and positive consequences of ICT use,” higher scores indicated greater negative and positive consequences, respectively; for “capacity to work using ICT,” higher scores indicated greater capacity; and for “negative and positive posture towards ICT use,” higher scores indicated more frequent adoption of negative and positive postures, respectively.

In addition to the questionnaire, sociodemographic data were collected, including age, sex, education level, employment situation, and company size. As it was an online questionnaire, some values were missing. To mitigate potential biases resulting from this, Little’s MCAR (Missing completely at Random) test was applied, determining that the missing data were completely random and did not pose a bias risk (*p* > 0.05).

### 2.4 Statistical analysis

Data analysis was conducted using the latest licensed version of the Statistical Package for the Social Sciences (SPSS). Percentages and frequencies were used to describe qualitative variables, while means, ranges, and standard deviations were used for quantitative variables. Statistically significant differences between results from 2016 and 2024 were sought using the Chi-square test, the Student’s *t*-test and the Mann–Whitney *U* test. Effect sizes were computed for all main comparisons. Cohen’s *d* was used for independent-samples *t* tests, and Rosenthal’s *r* was used for Mann–Whitney U tests. For chi-square tests, *φ* (2 × 2 tables) or Cramer’s V (larger tables) was reported; odds ratios (OR) with 95% confidence intervals were provided for selected dichotomous outcomes.

## 3 Results

### 3.1 Sociodemographic characteristics of teleworkers

Of all the teleworkers included in the sample, 64.4% (*n* = 488) responded to the questionnaire in 2016 and 35.6% (*n* = 270) in 2024. The mean age was 43.97 (±9.56) years. Gender participation was relatively balanced, with 55.1% women (*n* = 418), 44.1% men (*n* = 334), and only 0.4% (*n* = 3) selecting the “other” option. The most frequent education level was a master’s degree or doctorate (49.1%, *n* = 372), and the majority of respondents were full-time employees (82.7%, *n* = 627). The most common company size was over 500 employees (46.2%, *n* = 350). (Insert Table 2 here).

**Table 2:** Sociodemographic characteristics of Spanish teleworkers included in the study.

Characteristic		*n*	%
Sex	Female	418	55.1
	Male	334	44.1
	Other	3	0.4
Education level	Incomplete primary studies	1	0.1
	Basic and higher secondary	36	4.7
	Non-university higher studies	32	4.2
	Diploma or technical engineering	155	20.4
	Bachelor’s or higher engineering	162	21.4
	Master’s or doctorate	372	49.1
Employment status	Self-employed	13	1.7
	Unemployed, seeking work	55	7.3
	Full-time employment	627	82.7
	Part-time employment	54	7.1
	Retired	7	0.9
Company size	Fewer than 5 employees	55	7.3
	5 to 20 employees	46	6.1
	21 to 100 employees	81	10.7
	101 to 250 employees	81	10.7
	251 to 500 employees	62	8.2
	More than 500 employees	350	46.2
	No response	20	2.6

Missing data in sex, employment status, and company size resulted from participants omitting this information. Missing values were handled using Little’s MCAR test, confirming no bias despite missing data. *n* = 758.

In both study years, most workers used ICT for professional (93.9% *vs.* 92.6%), academic (56.4% *vs.* 63.0%), family (64.8% *vs.* 57.0%), and leisure purposes (79.5% *vs.* 71.1%). Although significant differences were observed in the family (*p* = 0.036) and leisure (*p* = 0.009) domains, the associated effect sizes were very small (*φ* = 0.076 and φ = 0.095, respectively). This indicates that, while statistically detectable, these changes represent only minor shifts in ICT use patterns between 2016 and 2024 rather than substantial behavioral transformations. The odds ratios suggest directional differences [family OR = 1.384, 95% IC (1.021, 1.876); leisure OR = 1.576, 95% IC (1.119, 2.221)], but their magnitude should be interpreted with caution given the small effect sizes. [Insert Table 3 here].

**Table 3:** Comparison of ICT usage domains among Spanish teleworkers in 2016 and 2024.

Domain		2016	2024	*p-*value*^a^	Effect size^b^	OR	95% IC
		*n* (%)	*n* (%)				
Professional	Yes	458 (93.9)	250 (92.6)	0.503	0.024	1.221	0.679–2.195
	No	30 (6.1)	20 (7.4)				
Academic	Yes	275 (56.4)	170 (63.0)	0.077	−0.064	0.759	0.560–1.030
	No	213 (43.6)	100 (37.0)				
Family	Yes	316 (64.8)	154 (57.0)	0.036	0.076	1.384	1.021–1.876
	No	172 (35.2)	116 (43.0)				
Leisure (recreation)	Yes	388 (79.5)	192 (71.1)	0.009	0.095	1.576	1.119–2.221
	No	100 (20.5)	78 (28.9)				

*For *p* < 0.05; ^a^Chi-square test (χ^2^), *df* = 1; ^b^Phi (φ) test. Odds ratios were calculated using 2024 as the reference category; therefore, OR > 1 indicates higher odds in 2016.

### 3.2 Personal experience of working with ICTs

Workers were asked whether different technologies caused them problems due to excessive use. In both years, workers generally reported that the Internet, social networks, video games, mobile phones, and television did not cause them problems; however, in 2024, more workers indicated that these technologies did cause them problems, compared to 2016. Increases in reported problems related to social networks (*p* < 0.001, *V* = 0.138,) and mobile phone use (*p* < 0.001, *V* = 0.121) reached statistical significance and showed small but consistent effect sizes. These findings suggest a modest shift toward perceiving certain digital tools as more problematic over time. However, other technologies showed either trivial effects or confidence intervals that included 1.00, indicating that not all observed differences represent robust changes in perceived technological burden. [Insert Table 4 here].

**Table 4:** Percentage of Spanish teleworkers reporting problems due to excessive use of different technologies in 2016 and 2024.

Device		2016	2024	*p-value* ^*a^	Effect size^b^
		*n* (%)	*n* (%)		
Internet	Yes	728 (14.8)	46 (22.3)	0.015	0.060
	No	416 (85.2)	160 (77.7)		
Social networks	Yes	50 (10.2)	58 (26.5)	< 0.001	0.138
	No	438 (89.8)	161 (73.5)		
Video games	Yes	12 (2.5)	13 (7.5)	0.003	0.092
	No	476 (97.5	161 (92.5)		
Mobile phone	Yes	77 (15.8)	70 (30.3)	< 0.001	0.121
	No	411 (84.2)	161 (69.7)		
Television	Yes	22 (4.5)	13 (7.5)	0.134	0.174
	No	466 (95.5)	161 (92.5)		

*For *p* < 0.05; ^a^Chi-square test (*χ*^2^), *df* = 1; ^b^Cramer’s *V* test.

When asked how they rated their personal experience with ICT, it was observed that those who rated it as quite negatively (0.2% *vs.* 0.8%), negative (1.3% *vs.* 3.6%), neutral (7.8% *vs.* 17.6%), and positive (40.6% *vs.* 46.0%) were more numerous in 2024. In contrast, the percentage of those who rated it as quite positive (36.1% *vs.* 22.0%) and very positive (14.0% *vs.* 10.0%) was lower in 2024. The distribution of evaluations shifted significantly (*p* < 0.001), and the effect size (*V* = 0.215) represents the largest association observed in the study, suggesting a meaningful though not large shift in overall subjective appraisal of ICT use.

### 3.3 Differences in technostress outcomes

For the subscale “negative implications of ICT use,” the mean score was 46.87 (±23.7) in 2016 and 48.85 (±21.4) in 2024, with a change in the median from 47.0 to 50.50. These results could suggest more negative implications between 2016 and 2024; however, the differences between the scores were not statistically significant (*p* > 0.050).

A significant difference was also identified in the score for “autonomy in the use of ICT,” decreasing from 13.68 (±4.4) in 2016 to 12.82 (±3.8) in 2024. The median decreased from 15.0 to 12.5 (*p* = 0.002). Effect size calculated using Rosenthal’s *r* test was |*r*| = 0.143, indicating a modest reduction in perceived control. This difference implies lower autonomy in aspects such as decision-making, time management, and skills in ICT use. Differences in autonomy scores were significant.

There was also a difference in the scores for “negative consequences of ICT use” between the years studied; 4.73 (±3.5) in 2016 and 5.95 (±3.1) in 2024 (*p* < 0.001). Effect size was |*r*| = 0.207, representing the largest change among the subscales, though still within the small-to-moderate rang. This difference implies that workers felt more concerned about responding to others via their devices and experienced greater time loss.

In line with this, differences in the score for “positive consequences of ICT use” was observed, the score was 5.39 (±2.9) in 2016 and 4.92 (±2.2) in 2024, with a median of 6.0 in 2016 and 5.0 in 2024. Effect size was |*r*| = 0.143, again a small effect. This supports the notion that ICT use has brought fewer positive consequences for workers.

On the other hand, the score for the capacity to work well using ICT was 17.5 (±6.9) in 2016 and 18.02 (±6.3) in 2024, with a statistically significant difference (*p* = 0.014). The median was 15 in 2016 and 19 in 2024. Effect size was |*r*| = 0.113, reflecting modest adaptation rather than a substantial improvement. This result supports the idea that workers have developed some more skills for using technologies, despite unexpected situations, problems, obstacles, and increased time and effort demands.

The average score for “positive personal posture” was 98.61 (±29.5) in 2016 and 95.8 (±28.2) in 2024. The median decreased from 102 to 96, although the difference was not significant (*p* = 0.214).

Finally, the average score for “negative personal posture” was higher in 2024 (35.42 ± 32.94) compared to 2016 (18.44 ± 19.90); this change was also reflected in the median, which rose from 15.00 to 35.00. The difference was confirmed to be significant (*p* < 0.001). Effect size was |*r*| = 0.230, indicating a moderate effect.

Overall, statistically significant differences were observed across several domains; however, effect sizes were generally small, indicating modest shifts rather than large-scale transformations. All the comparisons can be consulted in table [Insert Table 5 here].

**Table 5:** Comparison of technostress subscale scores among Spanish teleworkers between 2016 and 2024.

Dimension	Year	*x̅* (s)	Median	p-value*	Effect size
Negative implications of ICT use	2016	46.87 (23.7)	47	379^a^	−086^c^
	2024	48.85 (21.4)	50.50		
Autonomy in ICT use	2016	13.68 (4.4)	13.6	002^b^	−143^d^
	2024	12.82 (3.8)	12.5		
Negative consequences of ICT use	2016	4.73 (3.5)	4	<001^b^	207^d^
	2024	5.95 (3.1)	6		
Positive consequences of ICT use	2016	5.39 (2.9)	6	002^b^	−143^d^
	2024	4.92 (2.2)	5		
Capacity to work well using ICT	2016	17.5 (6.9)	15	014^b^	113^d^
	2024	18.02 (6.3)	19		
Positive personal posture	2016	98.61 (29.5)	102	214^b^	−061^d^
	2024	95.8 (28.2)	96.00		
Negative personal posture	2016	18.44 (19.90)	15.00	<001^b^	230^d^
	2024	35.42 (32.94)	35.00		

*For *p* < 0.050; ^a^Student’s *t*-test for independent samples. Kolmogorov–Smirnov normality test was applied to confirm normal distribution (*p* > 0.050). Homoscedasticity analysis was performed with Levene’s test (*p* < 0.050).

^b^Mann–Whitney *U* test; *df* = 1.

^c^Cohen’s *d* test; ^d^Rosenthal’s *r* test.

## 4 Discussion

This study provides novel comparative data on technostress trends among Spanish teleworkers. The scale used was specifically developed for the second and third editions of the Technostress Observatory. Similar instruments have not been used in the literature, limiting result comparisons. Nonetheless, some similarities and differences can be discussed.

The findings indicate temporal changes in technostress-related perceptions among Spanish teleworkers, but their magnitude suggests gradual adaptation processes coexisting with incremental stress-related demands rather than a structural escalation of technostress. The results indicate that most workers use ICT in professional, academic, family, and leisure contexts. Comparing measurements from 2016 and 2024, there were significant differences in the percentage of ICT use in the family domain (64.8% *vs.* 57.0%, *p* = 0.036) and leisure domain (79.5% *vs.* 71.1%, *p* = 0.009). Although this significant differences, the small effect sizes indicate that these variations likely reflect minor behavioral adjustments rather than meaningful lifestyle changes.

In contrast, perceptions of certain technologies—particularly social networks and mobile phones—as problematic increased with small but consistent effect sizes, suggesting a gradual intensification of perceived digital burden in specific areas. Despite the fact that most workers believe that ICT does not cause them problems, a modest change was observed in the percentage of those stating otherwise when using social networks (10.2% *vs.* 26.5%, *p* < 0.001), and mobile phones (15.8% *vs.* 30%, *p* < 0.001). Other studies report more excessive ICT use among men, although this difference was not significant (*p* = 0.875) (Villavicencio-Ayub et al., 2020), and it is more frequent in other Spanish-speaking countries like Mexico and Colombia (Quiroz González et al., 2023).

In the specialized technostress literature, “techno-addiction” is described as a variable linked to the excessive use of digital devices, capable of interfering with workers’ productivity and concentration, as well as with their personal and work relationships (Martín Rodriguez, 2020; Quiroz González et al., 2023; Villavicencio-Ayub et al., 2024). Although techno-addiction was not directly measured, it is possible to notice a reduction in ICT use in family and leisure contexts, while academic use increased and professional use remained stable. This pattern of ICT use does not suggest a widespread escalation of problematic overuse. Disinterest in ICT use has also been observed in other countries, such as Venezuela, where Cárdenas and Bracho (Cárdenas and Bracho, 2020) reported greater disinterest among administrative workers.

Other relevant findings of this study include a significant but small in magnitude reduction in the score for autonomy in ICT use (*x̄* = 13.68 *vs.* 12.82, *p* = 0.002), as well as in the perception of positive consequences of its use (*x̄* = 5.39 *vs.* 4.92, *p* = 0.002). Conversely, there was a difference in the perception of negative consequences of ICT use (*x̄* = 4.73 *vs.* 5.95, *p* < 0.001) and in the capacity to work well using ICT (*x̄* = 17.5 *vs.* 18.02, *p* = 0.014). These findings suggest a subtle erosion of perceived control and a modest rise in stress-related outcomes rather than a marked deterioration of working conditions. At the same time, the slight increase in capacity to work with ICT points toward adaptive learning processes that coexist with these stressors. These results are possibly a consequence of the inherent characteristics of teleworking; for example, long-term use of ICTs and the absence of clearly defined schedules could lead to reduced autonomy in organizing working time.

Additionally, the literature on technostress also mentions terms such as “techno-fatigue” and “techno-anxiety,” referring to excessive exhaustion and concern about teleworking outcomes, respectively. These situations could be present in the study sample and explain the results obtained. Technostress and techno-anxiety generate greater mental workload, as observed in this sample, and can lead to more negative consequences and fewer positive consequences from ICT use, which in turn imply work overload and restrictions for teleworkers. In the Ecuadorian context, this mental workload has already been associated with an increase in overall technostress and similar results have been obtained (Carrillo-Silva and Rodríguez-Llerena, 2024).

The inability to work well using ICT has been studied in other cases (Martín Rodriguez, 2020); for example, in a comparative study between Spanish and Portuguese populations, greater inefficiency was observed in the former, although not statistically significant (Maza, 2024). It is also typically higher in younger populations compared to older populations (*p* = 0.010) (Sánchez-Gómez et al., 2020). These results contrast with those of the present research, as a small but consistent difference in the capacity to work well using ICT was observed, which could be explained by the improvement of work skills during the pandemic period.

The findings suggest the need for preventive psychosocial interventions, targeted training programmes, and policy adjustments to ensure sustainable teleworking environments in Spain. Given the small effect sizes, preventive interventions should be framed not as responses to a crisis but as measures to manage a progressive and cumulative psychosocial load associated with prolonged ICT exposure. Policies targeting workload regulation, digital disconnection, and organizational support may help prevent the amplification of these modest but consistent trends over time. Within companies, training programmes and psychosocial support should help to mitigate the effects of technostress through preventive measures and early intervention.

Finally, it is important to mention three limitations of this study. Firstly, the use of self-report subscales implies the possibility of biases that could affect the validity of the results due to the subjective nature of the instrument, which depends on respondents’ perceptions. For example, the self-report subscales may involve social desirability bias which, in the present study, could translate into worker responses that do not truly reflect their actual perceptions, but rather aim to project a favorable self-image (in this case, as resilient workers able to adapt to the use of ICT without difficulty).

Secondly, the different number of participants in the two editions of the Technostress Observatory prevented a prospective study from being conducted. Future studies should incorporate qualitative analyses and explore sector-specific differences to deepen understanding of technostress dynamics.

Third, the rapid technological advances that occurred between 2016 and 2024, together with the increased availability and complexity of digital tools, may currently have mitigated the negative consequences of technostress (for example, through the use of AI to reduce workers’ cognitive load).

Despite the above limitations, the present study is considered to make theoretical contributions to the study of technostress in the post-pandemic context. First, it examines an already consolidated phenomenon, namely teleworking; second, it provides evidence on the trend observed among Spanish workers toward better adaptation to the use of ICTs, alongside a simultaneous increase in the negative consequences they perceive from such use. These findings support theoretical approaches that conceive technostress as a dynamic phenomenon in which the development of technological competences can coexist with heightened cognitive and organizational demands that generate stress in workers.

## 5 Conclusion

In conclusion, technostress assessments changed significantly between 2016 and 2024, but the magnitude of these changes was generally small. Though the data do not support the idea of a dramatic escalation of technostress after the pandemic, they indicate a pattern of incremental change, where improved digital competence occurs alongside a gradual rise in perceived cognitive and organizational demands. In this context, technostress appears to be evolving in intensity rather than undergoing a structural shift. This supports the conceptualization of technostress as a dynamic phenomenon characterized by parallel processes of skill adaptation and increasing psychosocial demands, with changes occurring progressively rather than abruptly. Workers reported slightly lower autonomy, fewer positive consequences, and more negative consequences of ICT use, alongside a modest increase in perceived capacity to use technology. These findings suggest gradual shifts in digital work experiences rather than large-scale transformations, highlighting the importance of ongoing preventive strategies to manage the cumulative effects of ICT-related demands. Regarding Spanish companies, interventions focused exclusively on enhancing workers’ skills in using ICTs may have little impact on technostress; companies should therefore direct their efforts towards factors such as workers’ autonomy. Future research should inform occupational safety policies to promote healthy teleworking conditions and could test variations of the research design used in this study, such as broadening the nationalities of teleworkers or collecting data over a longer period of time.

## Data Availability

The raw data supporting the conclusions of this article will be made available by the authors, without undue reservation.
